# On-Water Surface Synthesis of Two-Dimensional Polymer Membranes for Sustainable Energy Devices

**DOI:** 10.1021/acs.accounts.4c00356

**Published:** 2024-08-10

**Authors:** Feng Ni, Zhiyong Wang, Xinliang Feng

**Affiliations:** † Department of Synthetic Materials and Functional Devices, Max Planck Institute of Microstructure Physics, Halle (Saale) 06120, Germany; ‡ Center for Advancing Electronics Dresden (cfaed) and Faculty of Chemistry and Food Chemistry, Technische Universität Dresden, Dresden 01062, Germany

## Abstract

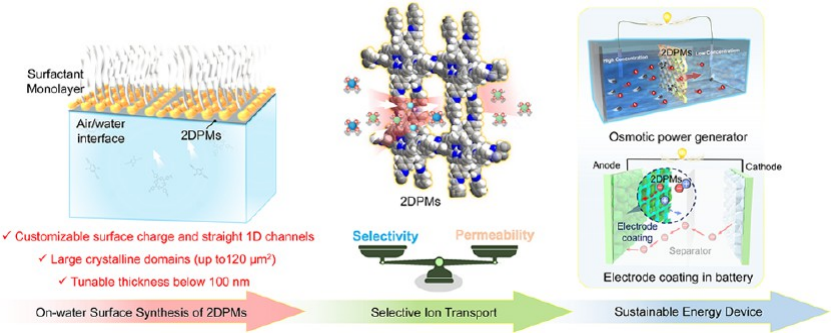

Ion-selective membranes are key components for sustainable energy devices, including osmotic power generators, electrolyzers, fuel cells, and batteries. These membranes facilitate the flow of desired ions (permeability) while efficiently blocking unwanted ions (selectivity), which forms the basis for energy conversion and storage technologies. To improve the performance of energy devices, the pursuit of high-quality membranes has garnered substantial interest, which has led to the exploration of numerous candidates, such as polymeric membranes (e.g., polyamide and polyelectrolyte), laminar membranes (e.g., transition metal carbide (MXene) and graphene oxide (GO)) and nanoporous 2D membranes (e.g., single-layer MoS_2_ and porous graphene). Despite impressive progress, the trade-off effect between ion permeability and selectivity remains a major scientific and technological challenge for these membranes, impeding the efficiency and stability of the resulting energy devices.

Two-dimensional polymers (2DPs), which represent monolayer to few-layer covalent organic frameworks (COFs) with periodicity in two directions, have emerged as a new candidate for ion-selective membranes. The crystalline 2DP membranes (2DPMs) are typically fabricated either by bulk crystal exfoliation followed by filtration or by direct interfacial synthesis. Recently, the development of surfactant-monolayer-assisted interfacial synthesis (SMAIS) method by our group has been pivotal, enabling the synthesis of various highly crystalline and large-area 2DPMs with tunable thicknesses (1 to 100 nm) and large crystalline domain sizes (up to 120 μm^2^). Compared to other membranes, 2DPMs exhibit well-defined one-dimensional (1D) channels, customizable surface charge, ultrahigh porosity, and ultrathin thickness, enabling them to overcome the permeability-selectivity trade-off challenge. Leveraging these attributes, 2DPMs have established their critical roles in diverse energy devices, including osmotic power generators and metal ion batteries, opening the door for next-generation technology aimed at sustainability with a low carbon footprint.

In this Account, we review our achievements in synthesizing 2DPMs through the SMAIS method and highlight their selective-ion-transport properties and applications in sustainable energy devices. We initially provide an overview of the SMAIS method for producing highly crystalline 2DPMs by utilizing the programmable assembly and enhanced reactivity/selectivity on the water surface. Subsequently, we discuss the critical structural parameters of 2DPMs, including pore sizes, charged sites, crystallinity, and thickness, to elucidate their roles in selective ion transport. Furthermore, we present the burgeoning landscape of energy device applications for 2DPMs, including their use in osmotic power generators and as electrode coating in metal ion batteries. Finally, we conclude persistent challenges and future prospects encountered in synthetic chemistry, material science, and energy device applications within this rapidly evolving field.

## Key References

- LiuK.; QiH.; DongR.; ShivhareR.; AddicoatM.; ZhangT.; SahabudeenH.; HeineT.; MannsfeldS.; KaiserU.On-water Surface Synthesis of Crystalline, Few-layer Two-dimensional Polymers Assisted by Surfactant Monolayers. Nat. Chem.2019, 11, 994–100010.1038/s41557-019-0327-531548668 .^1^*In this work, we developed the SMAIS method for the synthesis of highly crystalline and large-area 2DPMs on the water surface.*
- WangZ.; ZhangZ.; QiH.; Ortega-GuerreroA.; WangL.; XuK.; WangM.; ParkS.; HennersdorfF.; DianatA.On-water Surface Synthesis of Charged Two-dimensional Polymer Single Crystals via The Irreversible Katritzky Reaction. Nat. Synth.2022, 1, 69–7610.1038/s44160-021-00001-4.^2^*In this work, we demonstrated the synthesis of cationic 2DPMs with a tunable thickness ranging from 2 to 30 nm and crystal domain sizes up to 120 μm*^*2*^*via the irreversible Katritzky reaction.*
- ZhangZ.; BhauriyalP.; SahabudeenH.; WangZ.; LiuX.; HambschM.; MannsfeldS. C. B.; DongR.; HeineT.; FengX.Cation-selective Two-dimensional Polyimine Membranes for High-performance Osmotic Energy Conversion. Nat. Commun.2022, 13, 393510.1038/s41467-022-31523-w35803906 PMC9270359.^3^*This work demonstrated fully crystalline imine-linked 2DPMs capable of combining excellent ion permeability and high selectivity for osmotic power generation*.
- SabaghiD.; WangZ.; BhauriyalP.; LuQ.; MoragA.; MikhailoviaD.; HashemiP.; LiD.; NeumannC.; LiaoZ.Ultrathin Positively Charged Electrode Skin for Durable Anion-intercalation Battery Chemistries. Nat. Commun.2023, 14, 76010.1038/s41467-023-36384-536765051 PMC9918723.^4^*This work demonstrated the use of ultrathin, cationic 2DPMs with high anion permeability and selectivity as a graphite electrode coating for addressing the interfacial chemistry issues in batteries*.

## 1 Introduction

**Figure 1:**
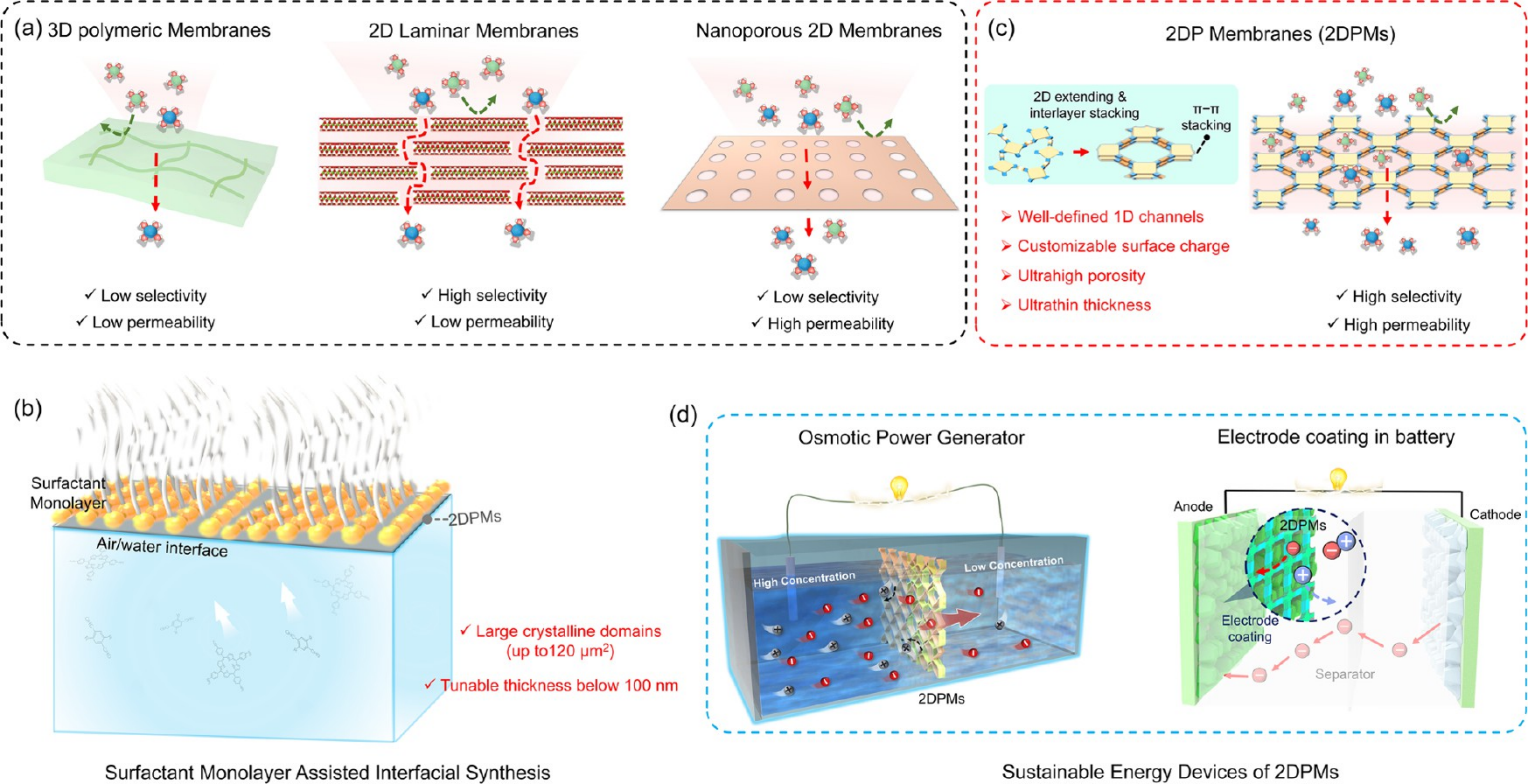
**Schematics of the selective-ion-transport properties, synthesis, and energy device applications of 2DPMs.** (a) Ion transport in representative membranes. (b) SMAIS strategy for synthesizing 2DPMs on the water surface. (c) Merits of 2DPMs for high ion selectivity and permeability. (d) 2DPMs applications in osmotic power generators and ion batteries.

Ion-selective membranes are integral to emerging energy devices, such as osmotic power generators,^5^ electrolyzers,^6^ fuel cells,^7^ and batteries.^8^ Driven by various gradients (e.g., pressure,^9,10^ electric field,^6^ concentration,^11^ temperature,^12^ etc.), these membranes selectively transport desired ions while chemically isolating unwanted processes, thereby critically influencing the performance metrics of energy devices, including efficiency and durability.^13^ Therefore, research into ion-selective membranes with high permeability necessitates meticulous design considerations.^11^ To date, diverse membranes have been developed to achieve efficient ion transport, including three-dimensional (3D) polymeric membranes (e.g., polyamide^14^ and polyelectrolyte^15^), two-dimensional (2D) laminar membranes (e.g., MXene^16^ and GO^17^), and nanoporous 2D membranes (e.g., single-layer MoS_2_^18^ and porous graphene^19^) (Figure 1a). However, 3D polymeric membranes suffer from low permeability and selectivity due to their disordered and broad-size-distributed channels.^20^ Conversely, 2D laminar membranes offer high selectivity, but their long ion transport pathways typically lead to limited ion permeability.^21^ Nanoporous 2D membranes provide ultrahigh permeability but often exhibit low ion selectivity due to insufficient functional groups.^22^ Therefore, mitigating the trade-off effect between ion permeability and selectivity remains a formidable challenge, limiting their application in sustainable energy devices.^23^

2D polymer membranes (2DPMs) are monolayer to few-layer porous polymeric materials with strict 2D planar periodicity.^24^ They can be synthesized either by exfoliation of bulk crystals of 2D COFs with subsequent assembly or by direct interfacial synthesis.^25^ In 2019, our group developed the surfactant-monolayer-assisted interfacial synthesis (SMAIS) method, which leverages the unique reactivity and selectivity of reactions to guide the preorganization and 2D polycondensation of rigid monomers on the water surface (Figure 1b).^1^ Recent investigations using SMAIS have successfully synthesized various highly crystalline 2DPMs, including quasi-2D polyaniline,^26^ 2D polyamide,^1^ 2D polyimide,^1^ 2D polyimine,^27,28^ 2D polyboronate ester,^29^ 2D poly(pyridinium salt),^2^ and vinylene-linked 2DPs,^30^ with tunable thicknesses from 1 to 100 nm and large crystalline domains up to 120 μm^2^. Compared to traditional membranes, these 2DPMs feature well-defined one-dimensional (1D) channels, customizable surface charge, ultrahigh porosity, and ultrathin thickness, making them promising platforms for addressing the trade-off effect (Figure 1c).^25,31^ Due to these attributes, 2DPMs have been successfully integrated into sustainable energy devices, including osmotic power generators^2,3,32^ and metal ion batteries,^4,30,33^ exhibiting excellent performance. Given the rapid progress in this field, a comprehensive review of recent advances, challenges, and future opportunities is crucial for integrating insights from chemistry, materials science, and energy device applications to guide future research.

This Account presents our progress in the synthesis of 2DPMs via the SMAIS method and their applications in sustainable energy devices. First, we systematically examine the SMAIS method as a means to fabricate highly crystalline 2DPMs on the water surface. In particular, the role of surfactant, the guided interaction between surfactant and monomer precursor, as well as the enhanced reactivity on the water surface will be highlighted. By analyzing critical parameters of 2DPMs, such as pore sizes, charged sites, crystallinity, and thickness, we then elucidate their structure–property relationships in selective ion transport. Additionally, we showcase the applications of 2DPMs in sustainable energy devices, including osmotic power generators and ion batteries (Figure 1d). Finally, we provide insights into persistent challenges in synthetic chemistry, material science, and energy devices that must be addressed to propel this field forward.

## 2 On-Water Surface Synthesis of 2DPMs through the SMAIS Method

Current methodologies for synthesizing 2DPMs employ either top-down or bottom-up strategies.^25^ Top-down strategies involve the physical and chemical delamination of layer-stacked 2DP bulk crystals (also known as 2D COFs), followed by filtration, casting, or reconstitution to form 2DPMs.^34^ However, these membranes, consisting of randomly stacked crystallites, often exhibit disordered and widely distributed channels, resulting in low ion permeability and selectivity. Conversely, bottom-up methods, including on-surface synthesis, liquid–liquid interfacial synthesis, and on-water surface synthesis (e.g., Langmuir–Blodgett (LB) method and SMAIS method), offer promising solutions to these challenges.^35,36^ On-surface synthesis facilitates direct growth of 2DPMs at the vapor–solid or liquid–solid interface, suitable for producing monolayer to few-layer 2DPMs.^37,38^ However, the strong molecule–substrate interactions typically result in limited domain sizes (less than 100 nm) and complicate membrane transfer for device integration. Alternatively, the liquid–liquid and LB methods, which provide a common platform for the synthesis of thick (few nm to μm) and monolayer 2DPMs, respectively, with easy transfer of resulting films.^39,40^ However, the stochastic directionality and large-amplitude motions of monomers, coupled with a lack of supramolecular forces driving preorganization into long-range ordered structures, often lead to 2DPMs with poor crystallinity and random layer orientation.

**Figure 2:**
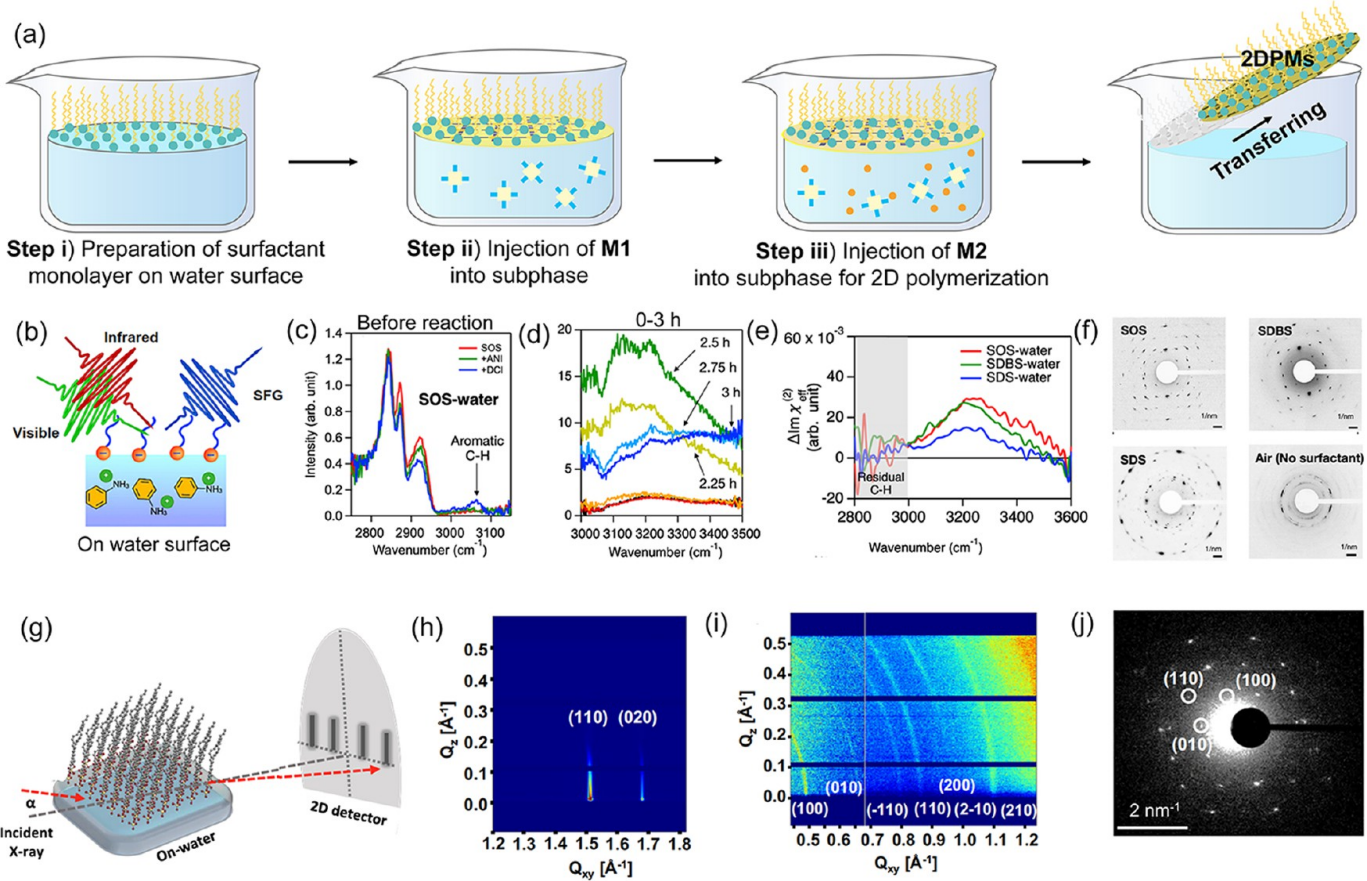
**On-water surface synthesis of 2DPMs via the SMAIS method.** (a) Schematic of the SMAIS procedure. (b) Illustration of the in situ SFG measurement. SFG spectra in the C–H stretch mode region recorded from the SOS-water interface (c) before polymerization and (d) within the first 3 h. (e) Difference in heterodyne-detected (HD) SFG spectra at 100 mM and 1 M salt concentrations. (f) SAED images of quasi-2D PANIs obtained from different surfactant–water interfaces. Adapted with permission from ref (43). Copyright 2021 Elsevier. (g) Illustration of the in situ GIWAXS measurements. GIWAXS patterns of (h) SOS monolayer in step i and (i) products after injecting **M1** in step ii. (j) SAED pattern of the transferred **M1**-assembly films in step ii. Reproduced from ref (44). Copyright 2023 Springer Nature.

The SMAIS method developed by our group in 2019 opens a door to tackle the aforementioned challenges.^1^ As illustrated in Figure 2a, the SMAIS method generally involves three steps: (i) spreading the molecular surfactant (e.g., sodium oleyl sulfate (SOS), stearic acid (SA), sodium dodecyl sulfate (SDS), sodium 4-dodecylbenzenesulfonate (SDBS), and cetyltrimethylammonium bromide (CTAB)) or polymeric surfactants (e.g., poly(acrylic acid)^41^ and poly(sodium 4-styrenesulfonate)^42^) on the water surface to form an organized floating monolayer with stable surface pressure. The selection of surfactants is guided by their potential interactions with monomer precursors. For instance, negatively (or positively) charged surfactants can induce electrostatic interactions with positively (negatively) charged monomers, facilitating their adsorption and preorganization on the water surface. Other guided interactions, such as hydrogen bonding, coordination bonds, and strong covalent bonds, are also considered based on the functional groups present in the monomers. (ii) Injecting an aqueous solution of monomer 1 (**M1**) causes the monomers to diffuse, be absorbed, and preorganize beneath the surfactant monolayer. (iii) Adding monomer 2 (**M2**) initiates 2D polymerization on the water surface.

To elucidate the role of surfactants and unravel the molecular-level mechanisms behind the synthesis of 2DPMs using the SMAIS method, we utilized in situ techniques such as sum frequency generation (SFG) spectroscopy and grazing incidence wide-angle X-ray scattering (GIWAXS) to monitor the assembly of monomers and the subsequent 2D polymerization and crystallization processes in real-time. For example, using SFG, we tracked the growth of quasi-2D polyaniline (PANI) on the water surface (Figure 2b).^43^ The variation in characteristic SFG spectra indicated the accumulation of monomers underneath the SOS monolayer driven by the electrostatic interaction (Figure 2c). Following the injection of ammonium persulfate, drastic changes in the SFG signal after 2 h suggested the occurrence of the reaction on the water surface (Figure 2d). Notably, the positively charged = NH_2_ groups are generated between 2 to 2.5 h and then self-organized on the water surface, guided by the hydrophilic sulfonic acid group of SOS. In this respect, the type of surfactants, providing different charge densities on the water surface, was crucial in defining the nucleation density and crystallinity of quasi-2D PANI. As shown in Figure 2**e**-**f**, surfactants with higher charge densities, such as SOS and SDBS resulted in quasi-2D PANI with higher crystallinity compared to SDS and the absence of surfactant.

In another study, we employed in situ GIWAXS to probe the crystal structure of surfactant and porphyrin-based monomers preorganization on the water surface (Figure 2g).^44^ The GIWAXS pattern of SOS on the water surface (step (i) revealed sharp and discrete Bragg spots near Q_Z_ = 0, indicating the formation of a long-range ordered SOS monolayer (Figure 2h). Upon adding **M1** (4-(5,10,15-triphenylporphyrin-20-yl)aniline) to the water subphase (step (ii), GIWAXS demonstrated epitaxial growth of **M1** with a self-organized superstructure underneath the SOS monolayer (Figure 2i). The selected-area electron diffraction (SAED) pattern of the self-organized **M1** film transferred onto the transmission electron microscope (TEM) grid matched that of GIWAXS, manifesting with smaller lattice parameters compared to individual **M1** molecules due to the unique 2D confinement in J-aggregation (Figure 2j). After adding **M2** (perylene-3,4,9,10-tetracarboxylic dianhydride) for 10 h, the SOS-**M1** assembly and J-aggregated confinement of **M1** remained intact (step (iii), directing the on-water reactions of **M2** with **M1** for crystallization. Furthermore, the in situ SFG technique suggested that this sequential assembly could further promote layer-to-layer growth of multilayer products through site-selective imide bond formation and simultaneous release of interfacial charges.

On the other hand, on-water surface reactions typically exhibit higher reactivity than those in aqueous solutions. To understand this behavior, we conducted model reactions and employed matrix-assisted laser desorption/ionization-time-of-flight mass spectrometry (MALDI-TOF MS) to monitor the evolution of the products.^2,30,44^ Take the Katritzky reaction as an example, model reactions between **M1** (5-(4-aminophenyl)-10,15,20-(triphenyl)porphyrin) and **M2** (2,4,6-triphenylpyrylium tetrafluoroborate) successfully yielded the targeted products on the water surface, in contrast to incomplete reactions and low conversion rates in aqueous solutions.^2^ Likewise, the Knoevenagel model reaction demonstrated enhanced reactivity and reversibility on the water surface compared to aqueous conditions.^30^ This enhanced reactivity can be attributed to oriented monomer assembly governed by intermolecular interactions, stabilization of the activated complex by hydrogen bonding,^2,30^ and the absence of solvation effects on the water surface.^45−47^ These results indicate that the SMAIS method not only facilitates supramolecular chemistry by preorganizing and interlocking precursor monomers on the water surface but also provides a 2D confined geometry that enhances the selectivity and reactivity for subsequent 2D polymerization.

**Figure 3:**
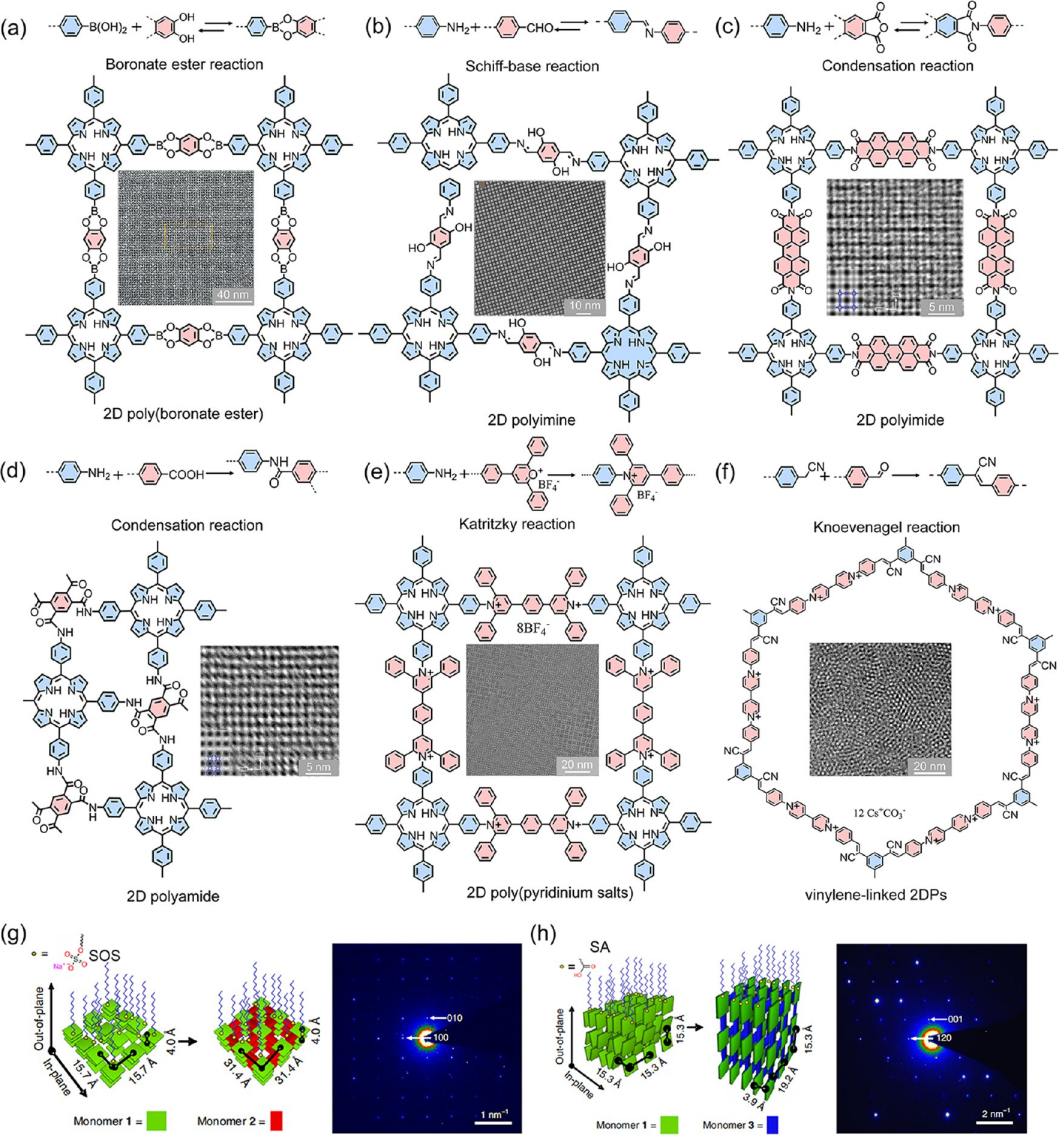
**The obtained 2DPMs via the SMAIS method.** (a-f) Representative synthetic chemistry and resultant products. (a) reproduced from ref (29). Copyright 2020 John Wiley and Sons. (b) reproduced from ref (49). Copyright 2020 AAAS. (c) and (d) reproduced from ref (1). Copyright 2019 Springer Nature. (e) adapted with permission from ref (2). Copyright 2021 Springer Nature. (f) reproduced from ref (30). Copyright 2024 John Wiley and Sons. Schematics of the resulting 2DPMs with (g) face-on and (h) edge-on orientations guided by different surfactants. Reproduced from ref (1). Copyright 2019 Springer Nature.

On this basis, we have utilized a range of synthetic chemistry, including dynamic covalent reactions (e.g., boronate-ester,^29^ Schiff-base,^27^ condensation reactions,^1^ and coordination reactions^48^) and kinetically irreversible reactions (e.g., Katritzky reactions^2^ and Knoevenagel reactions^30^), for the synthesis of 2DPMs through the SMAIS method (Figure 3). These membranes are free-standing with large-area (∼28 cm^2^), preferential layer orientation and large crystal domain up to 120 μm^2^. By adjusting the monomer concentrations and reaction time, the thickness of 2DPMs can be tuned from 1 to 100 nm. Furthermore, by carefully modulating the guided interactions between monomers and surfactants on the water surface, we can precisely control the preferential layer orientations of these 2DPMs, shifting between face-on and edge-on configurations.^1^ For instance, the SOS surfactant monolayer enables the parallel adsorption of **M1** (4,4′,4′′,4′′′-(porphyrin-5,10,15,20-tetrayl)tetraaniline) through electrostatic interaction and hydrogen bonding, creating a face-on arrangement (Figure 3g). SA that possesses −COOH headgroup reacts with one amine group to form a covalent amide bond, allowing **M1** to be vertically anchored beneath the SA monolayer (Figure 3h). Upon adding **M2** (1H,3H-Furo[3,4-*f*][2]benzofuran-1,3,5,7-tetrone), the preferential face-on or edge-on growth of 2DPMs was achieved on the water surface.

## 3 Selective-Ion-Transport Properties of 2DPMs

**Figure 4:**
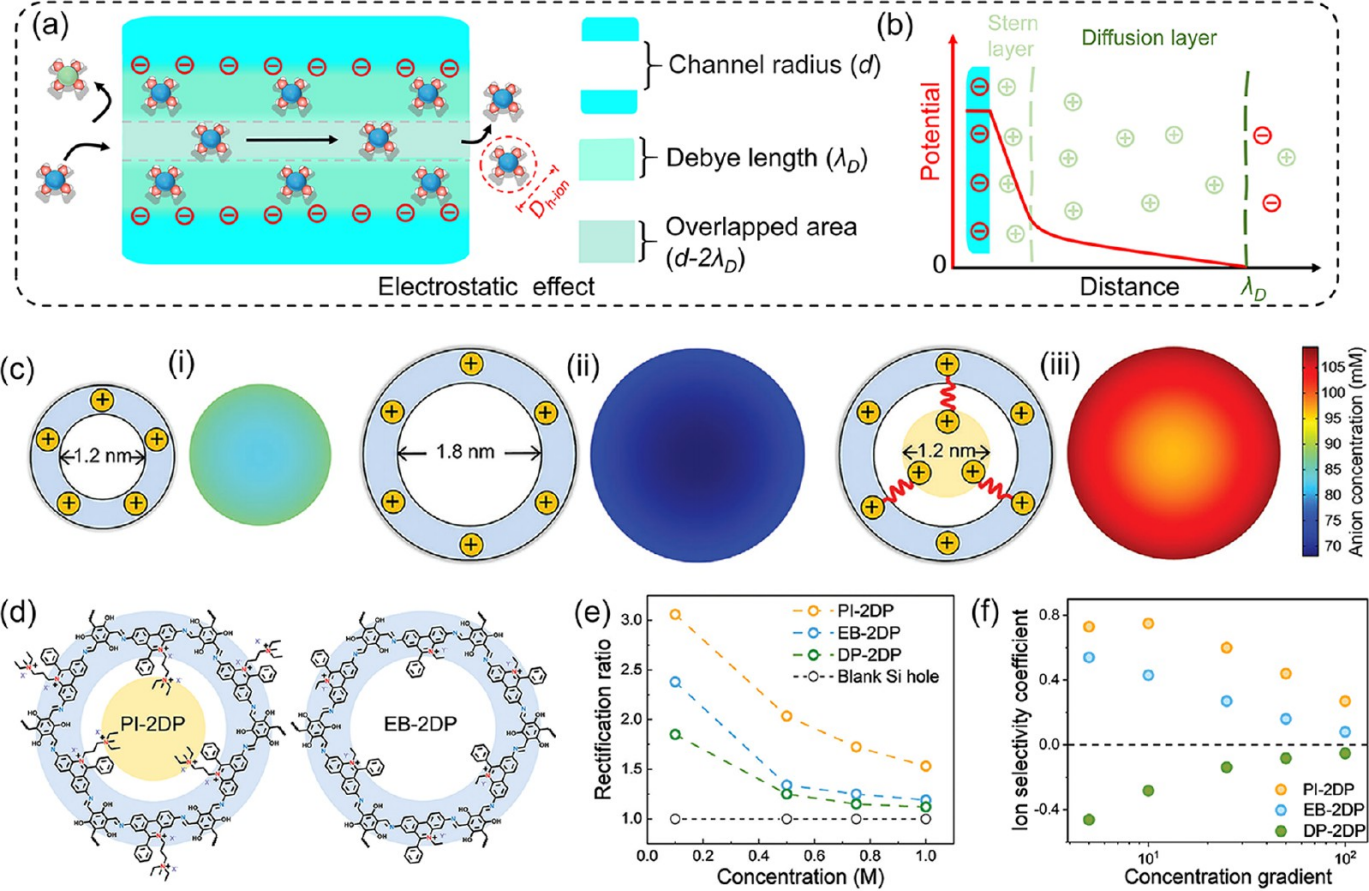
**Engineering pore size for enhanced selective-ion-transport properties.** (a) Schematics of electrostatic-effect ion transport in 2DPMs. (b) Potential field distribution within the EDL of 2DPMs, consisting of a first Stern layer and a second diffusion layer. (c) Simulated anion concentration profiles within 1D channels with varying *d* values. (d) Chemical structures of PI-2DP and EB-2DP. (e) Rectification ratios of PI-2DP and EB-2DP at different KCl concentrations. (f) Ion selectivity coefficient of PI-2DP and EB-2DP in increasing KCl concentration gradients. Reproduced from ref (32). Copyright 2024 John Wiley and Sons.

The ion transport within 2DPMs is primarily dominated by size and electrostatic effect, operating across different length scales. The size effect leverages narrow nanochannels (typically less than 8.5 Å) to sieve preferential ions based on physical size exclusion.^22,50^ However, achieving Å-level precision in pore size modulation to selectively separate ions with different hydrated diameters remains challenging in current 2DPMs. Conversely, efficient selective ion transport can be achieved via electrostatic effects (Figure 4a).^11,22^ When immersed in electrolyte solutions, the charged ionic groups of 2DPMs form an electrical double layer (EDL).^51^ As the channel radius (*d*) approaches the thickness of the EDL, overlapping EDLs result in electrostatic interactions that preferentially transport counterions over co-ions along the diffusion layer, imparting ion selectivity (known as surface-charge-governed ion transport).^52^ The thickness of the resulting EDL is quantified by the Debye length (λ_*D*_), which is defined as^53^
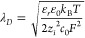
where ε_*r*_ and ε_0_ refer to the relative and vacuum permittivity, respectively, *k*_B_ is the Boltzmann constant, *T* is the absolute temperature, *z*_*i*_*, c*_*0*_ and *F* are the valence number, the concentration of the solution and the Faraday’s constant, respectively. As illustrated in Figure 4b, the potential distributions within channels dictate the concentration of transported ions to achieve ion selectivity, which could, in turn, affect their ion permeability. Engineering the pore sizes of 2DPMs thus provides a direct method to modulate selective ion transport. Our recent study investigated the impact of channel sizes on ion transport properties in 2DPMs.^32^ As shown in Figure 4c, numerical simulations indicate higher anion concentration and electric potential in narrower charged pores due to more overlapped EDLs. Encouraged by these insights, we synthesized two types of cationic 2DPMs with similar thicknesses (12–14 nm): propidium iodide-based 2DP membranes (PI-2DP, pore size: 1.2 nm) and ethidium bromide-based 2DP membranes (EB-2DP, pore size: 1.8 nm) (Figure 4d). Ion transport measurements revealed more overlapped EDLs in PI-2DP, resulting in higher anion (e.g., Cl^–^) selectivity coefficients (∼0.8) (Figures 4e-f). These results underscore the importance of designing narrower pore channels to enhance the ion selectivity of 2DPMs.

**Figure 5:**
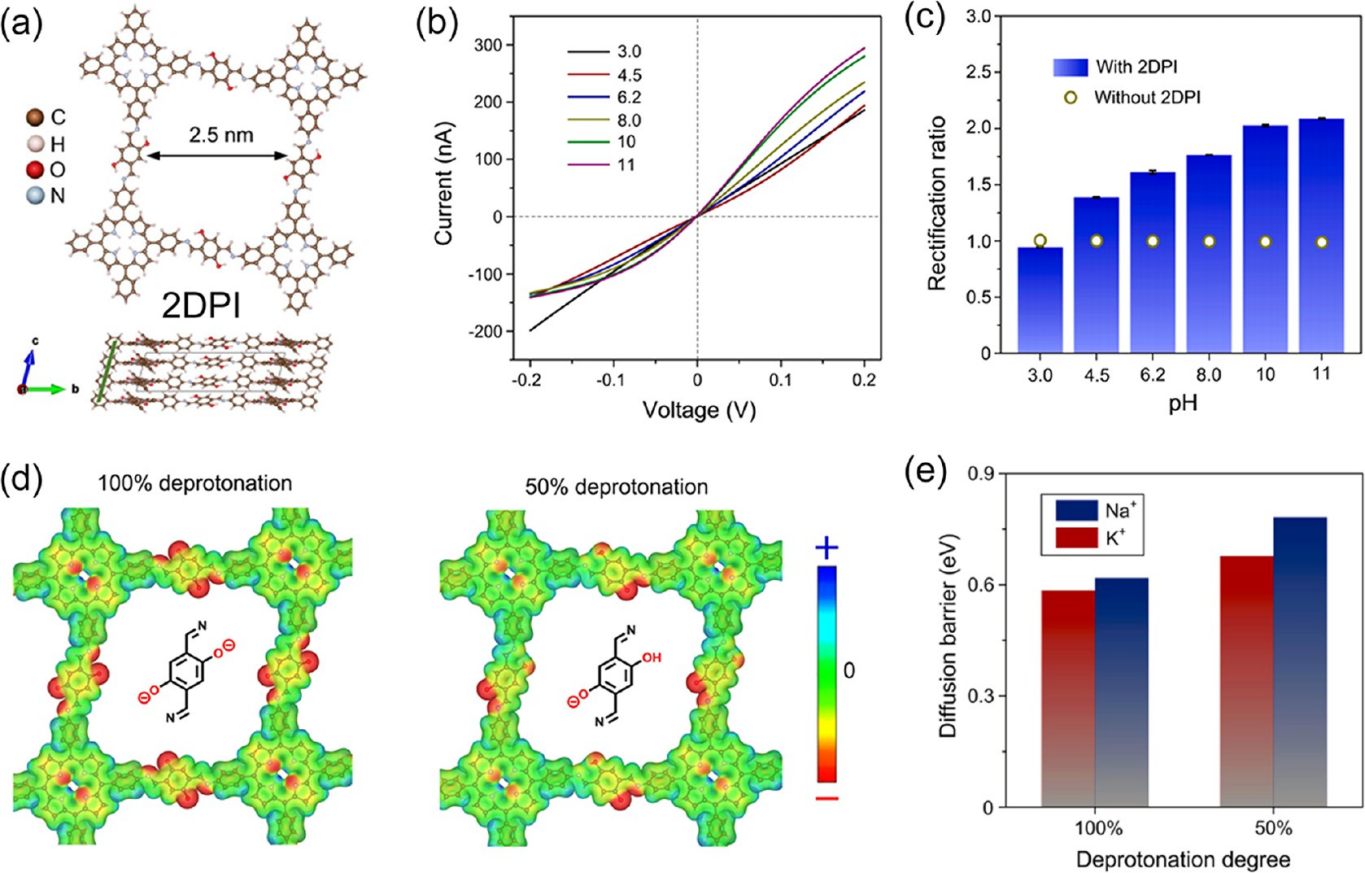
**Selective-ion-transport properties of 2DPMs governed by ionic groups.** (a) Structure of 2DPI. (b) I–V curves and (c) rectification ratios of 2DPI in various electrolytes with rising pH values. (d) Electrostatic potential (ESP) surface and (e) diffusion barriers of fully deprotonated (100%) and partially (50%) deprotonated 2DPI. Reproduced from ref (3). Copyright 2022 Springer Nature.

Apart from pore sizes, ionic groups that define charge polarity and density in 2DPMs are crucial for their selective-ion-transport properties.^11^ Typically, anionic (e.g., hydroxyl, carboxyl, and sulfonic) and cationic groups (e.g., amino, pyridinium, and ethidium) with distinct dissociation constants (p*K*_a_) become charged upon deprotonation or protonation, resulting in negatively or positively charged surfaces.^3,32,54−57^ This charge polarity determines which type of ions can be selectively transferred. For example, negatively charged pore channels facilitate the selective transport of cations, while positively charged ones enable anions transport. Incorporating more ionic groups during 2DPM synthesis enhances charge density, leading to greater potential distributions and thicker EDL within 2DPMs, thereby improving ion selectivity.^58^ Recently, our studies attempted to tune the charge density of 2D polyimine (2DPI) by controlling the pH of the electrolytes (i.e., KCl and NaCl) (Figure 5a).^3^ Ion transport in 2DPI was found to be pH-dependent, showing significant ionic rectification at high pH due to increased surface charge densities from hydroxyl group deprotonation (Figures 5b-**c**). Afterward, a fully deprotonated 2DPI exhibited lower barriers for K^+^ and Na^+^ ions than the partially deprotonated one (Figures 5d-e). This is attributed to the ions binding more readily to available active hydroxyl groups, enhancing their diffusion within the 2DPI.

**Figure 6:**
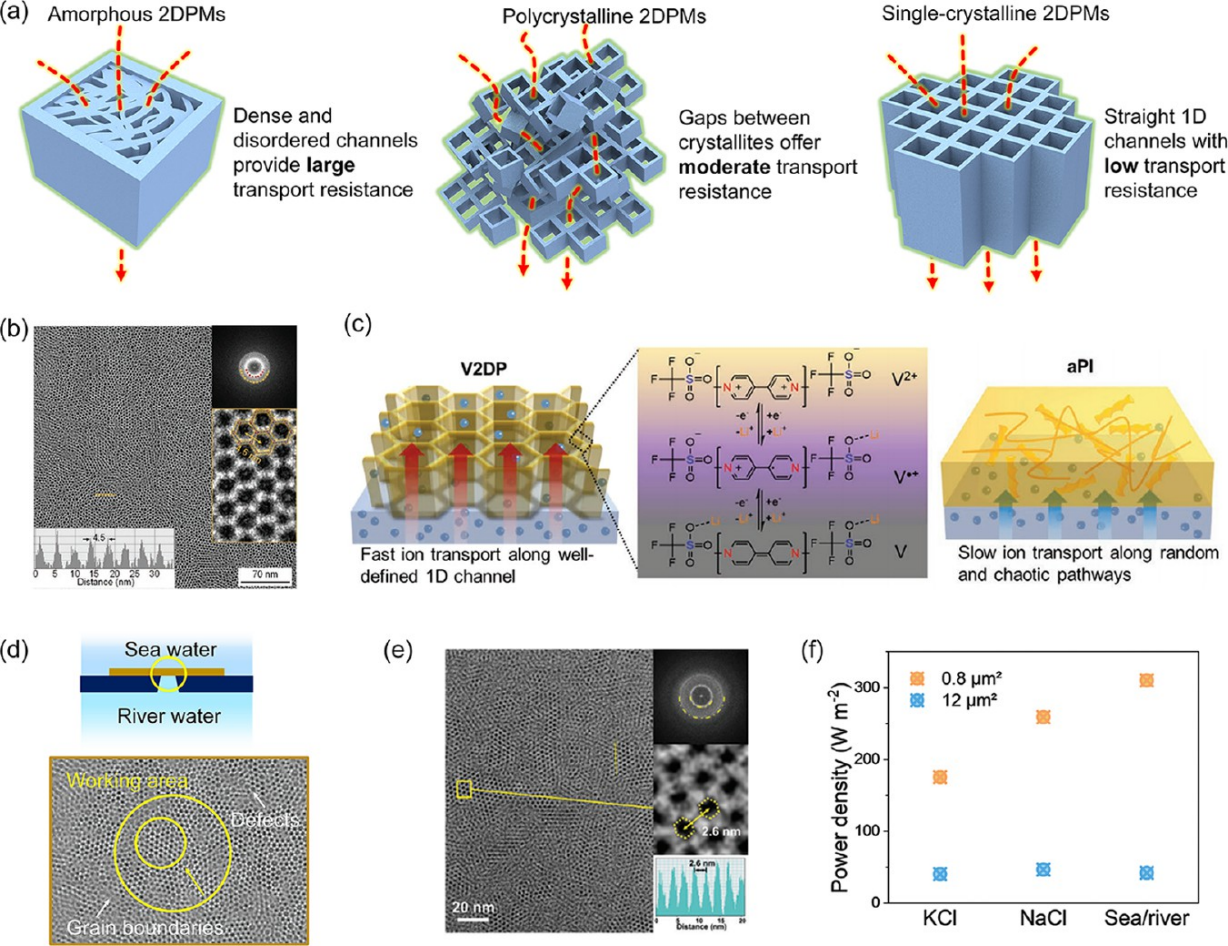
**The impact of 2DPM crystallinity on selective-ion-transport properties.** (a) Schematic comparison of ion transport in amorphous, polycrystalline, and single-crystalline 2DPMs. (b) High-resolution TEM (HRTEM) image of V2DP with SAED pattern (inset). (c) Schematic of ion transport pathways within V2DP and amorphous polyimine (aPI). Adapted with permission from ref (60). Copyright 2021 John Wiley and Sons. (d) Illustrations of the working area of polycrystalline 2DPMs for ion-transporting testing. (e) HRTEM image of polycrystalline PI-2DP with SAED pattern (inset). (f) The power density of PI-2DP with varying working areas. Reproduced from ref (32). Copyright 2024 John Wiley and Sons.

The crystallinity (i.e., crystalline domain and layer orientation) of 2DPMs reflects how long-range-ordered their inherent 1D channels are, essential for achieving both high ion selectivity and permeability. In amorphous 2DPMs and polycrystalline 2DPMs with random layer orientation, disordered channels create barriers for ion transport, resulting in low permeability (Figure 6a).^59^ On the other hand, wide-size-distributed channels in amorphous 2DPMs and the gaps between the crystal boundaries in polycrystalline 2DPMs may compromise ion selectivity. For example, fully crystalline and face-on oriented 2DPMs like viologen-immobilized 2DP membranes (V2DP) demonstrate faster ion transport along well-defined 1D channels due to less tortuous paths and lower ion diffusion resistance (Figure 6b-c).^60^ Furthermore, polycrystalline 2DPMs contain numerous grain boundaries and defects, significantly impacting their selective-ion-transport properties. To assess this influence, we conducted contrast experiments by varying the working area of polycrystalline 2DPMs (Figure 6d). For example, with PI-2DP, reducing the working area from 12 to 0.8 μm^2^ greatly improved ion selectivity, leading to increased output power density from 42.2 to 310 W m^–2^ (Figures 6e-f).^32^ This improvement is attributed to minimized penetration resistances from grain boundaries and defects. These findings emphasize the potential of achieving large single crystals to maximize the selective-ion-transport properties of 2DPMs.

**Figure 7:**
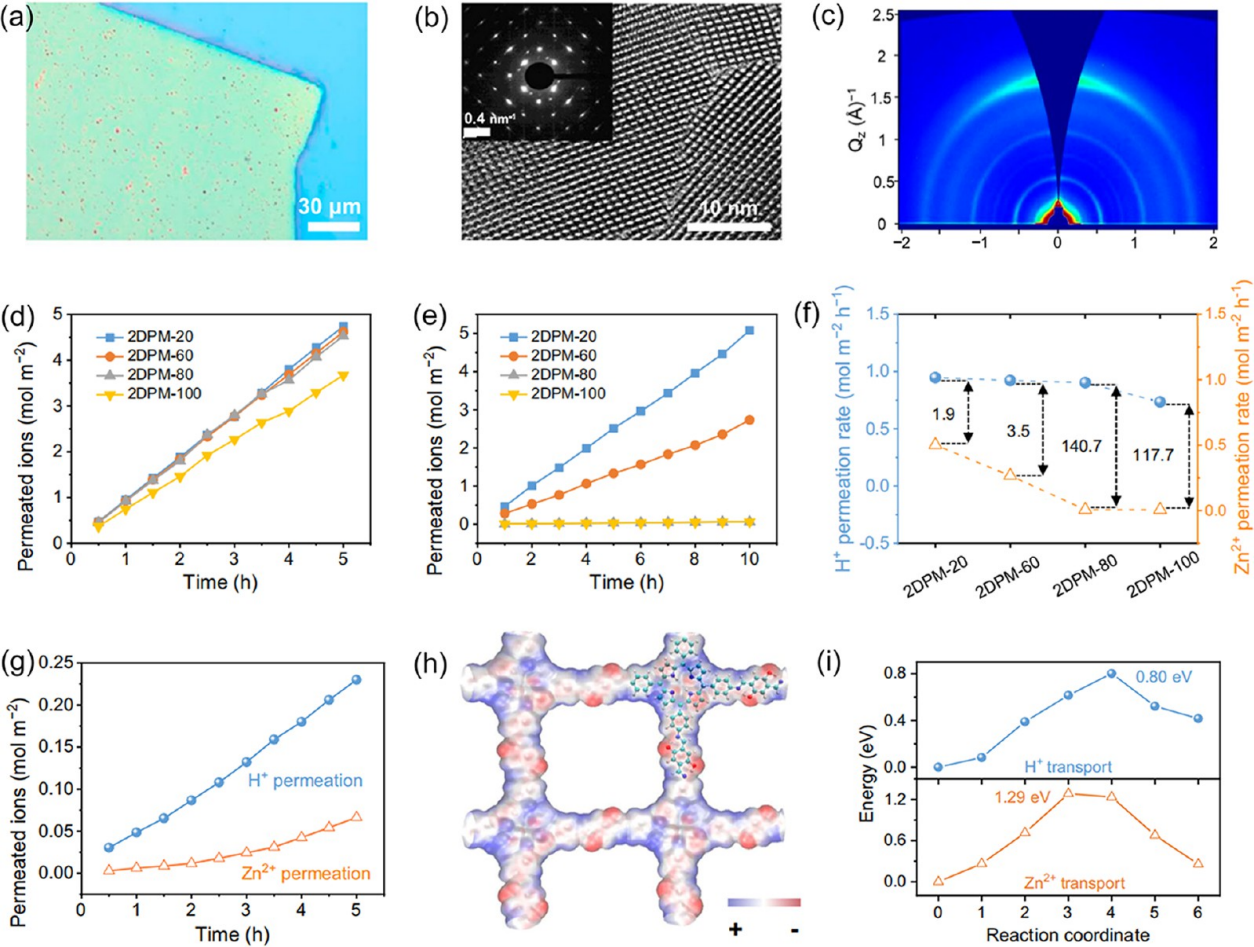
**Modulation of selective-ion-transport properties in 2DPMs by thickness.** (a) Optical microscopy image, (b) HRTEM image with inset SAED image, and (c) GIWAXS pattern of 2DPI. (d) H^+^ and (e) Zn^2+^ permeation curves, and (f) H^+^/Zn^2+^ selectivity of various 2DPI membranes with different thicknesses ranging from 20 to 100 nm. (g) H^+^ and Zn^2+^ permeation curves of 2DPI-80 in 2 M ZnSO_4_. (h) ESP surface of 2DPI. (i) Corresponding energy profiles of H^+^ and Zn^2+^ through the 2DPI. Reproduced from ref (33). Copyright 2024 Springer Nature.

Ultrathin 2DPMs (<100 nm) offer short ion transport pathways with low barriers, crucial for enhancing ion permeability while maintaining high selectivity.^13^ Leveraging the high crystallinity and face-on orientation of 2DPI, we investigated a novel thickness-dependent H^+^/Zn^2+^ selectivity (Figure 7a-c).^33^ By varying the thickness from 20 to 100 nm, 2DPI exhibited a linear permeation relationship with constant transport rates for both H^+^ and Zn^2+^, with Zn^2+^ exhibiting a stronger thickness-dependent transport behavior (Figure 7d-e). Consequently, the 80 nm-thick 2DPI (2DPI-80) demonstrated the highest H^+^/Zn^2+^ selectivity of 140.7 while retaining a high H^+^ permeation rate (Figure 7f). Even in a typical electrolyte ((i.e., 2 M ZnSO_4_, pH = 4.3, C_Zn_^2+^/C_H_^+^ = 4 × 10^4^), 2DPI-80 exhibited an enhanced H^+^ permeation rate (0.046 mol m^–2^ h^–1^) compared to Zn^2+^ (0.013 mol m^–2^ h^–1^), achieving an excellent H^+^/Zn^2+^ selectivity of 3.5 (Figure 7g). This superior selectivity arises from enriched favorable sites in 2DPI facilitating H^+^ transport, including the O atoms of phenolic hydroxyl groups, N atoms of imine bonds, and more accessible porphyrin pyrrole units (Figure 7h). In contrast, Zn^2+^ transport is limited to phenolic hydroxyl and imine groups and requires higher transport energy (1.29 eV vs. 0.8 eV of H^+^) (Figure 7i). These findings support the experimental observation of the thickness-dependent selectivity of 2DPI.

## 4 Applications of 2DPMs in Sustainable Energy Devices

The exceptional ion selectivity and permeability of 2DPMs make them highly promising for sustainable energy devices requiring high-performance ion-selective membranes. This section will examine our recent advancements in the use of 2DPMs in osmotic power generators and as electrode coatings in metal ion batteries.

### 4.1 Osmotic Power Generators

**Figure 8:**
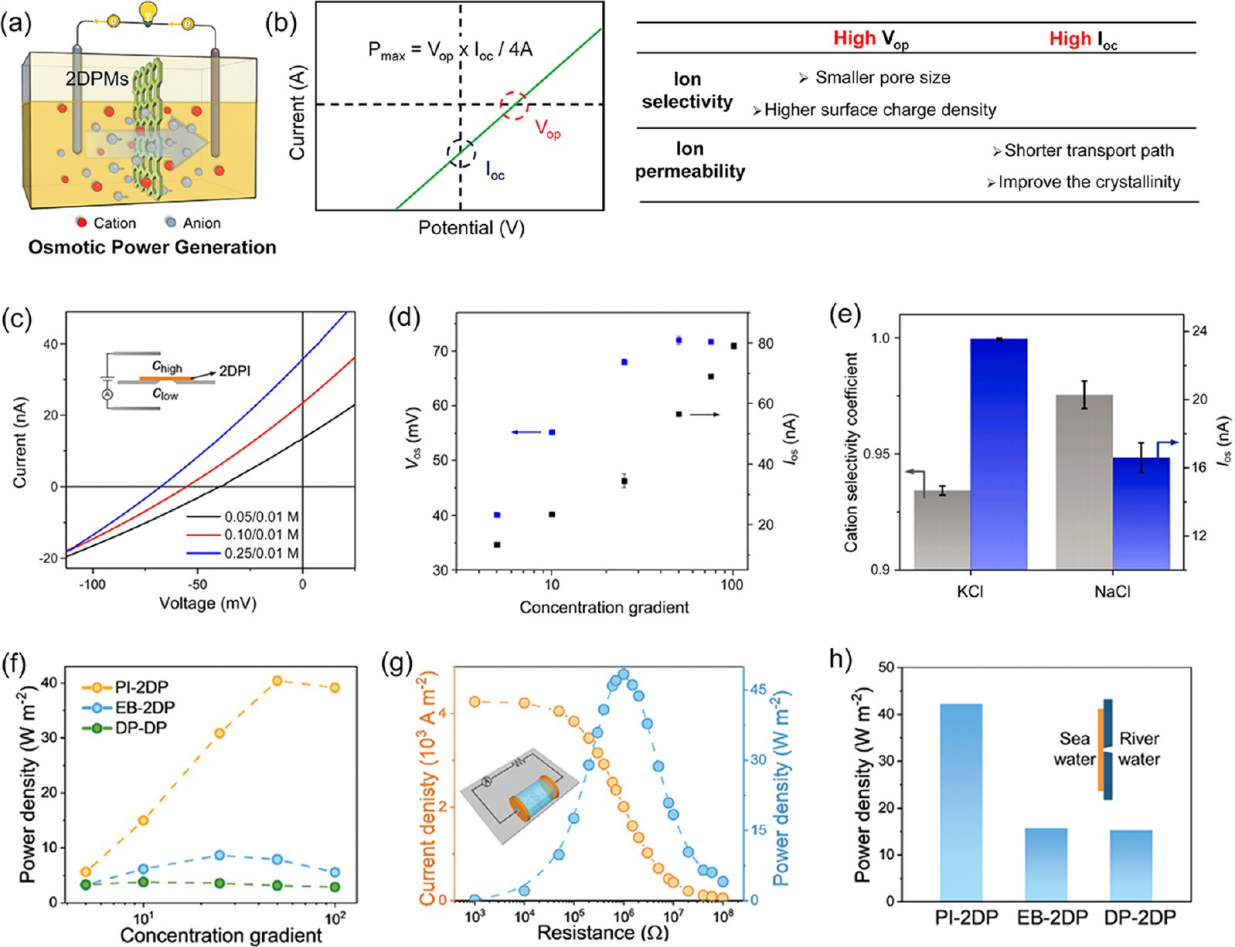
**Application of 2DPMs in osmotic power generators.** (a) Schematic depicting osmotic power generation using 2DPMs. Reproduced from ref (32). Copyright 2024 John Wiley and Sons. (b) I–V curve measured for osmotic power generation and guidelines for enhancing *V*_*op*_ and *I*_*oc*_ in 2DPMs. (c) Measured I–V curves and (d) *V*_*op*_ and *I*_*oc*_ values of osmotic power generators using 2DPI at varying KCl gradients. (e) Cation selectivity coefficient and *I*_*oc*_ of the 2DPI-based system at 10-fold KCl and NaCl. Reproduced from ref (3). Copyright 2022 Springer Nature. (f) The *P*_*max*_ of PI-2DP under different KCl gradients. The *P*_*out*_ of PI-2DP on the external circuit *I*_*oc*_ (g) under an artificial 50-fold NaCl gradient and (h) by mixing natural river water and seawater. Reproduced from ref (32). Copyright 2024 John Wiley and Sons.

Harvesting blue energy, or osmotic power, via reverse electrodialysis technologies presents substantial potential for large-scale electricity supply in both industrial and domestic contexts.^21^ Typically, this involves an ion-selective membrane separating saltwater and freshwater reservoirs, facilitating the selective transport of cations or anions. This method utilizes the Gibbs free energy released during solution mixing for power generation.^21^ The development of 2DPMs offers an appealing opportunity to achieve optimal osmotic power density due to their superior selective-ion-transport properties. In this regard, the performance of osmotic power conversion is assessed using an electrochemical cell under various salt concentration gradients across the 2DPMs (Figure 8a). By measuring the open-circuit voltage and short-circuit current, the osmotic potential (*V*_*op*_) and osmotic current (*I*_*oc*_) can be determined, allowing the calculation of theoretical maximum power density (*P*_*max*_) using the equation: *P*_*max*_ = *V*_*op*_*× I*_*oc*_*/4A*, where *A* is the working area (Figure 8b). Therefore, improving the *V*_*op*_ and *I*_*oc*_ values, influenced by the ionic selectivity and permeability of 2DPMs, can significantly increase power output.

For instance, we developed a fully crystalline 2D polyimine (2DPI) with a 70 nm thickness for osmotic power generation, leading to devices with significant open-circuit voltage (V_op_) and short-circuit current (I_oc_) values across different KCl concentration gradients (Figure 8c).^3^ The measured *I*_*oc*_ increases from 5 to 100, while the *V*_*op*_ initially rises from 40 to 72 mV before declining at concentration gradients exceeding 50 (Figure 8d). Moreover, 2DPI demonstrates ultrahigh cation selectivity coefficients of 0.98 and 0.93 at 5-fold NaCl and KCl gradients, respectively, along with high ionic flux (Figure 8e). As a result, the *P*_*max*_ of 2DPI reached 20.3 and 27.6 W m^–2^ in 10-fold NaCl and KCl gradients, respectively, surpassing other 2DPMs.^37,54^ Additionally, we have presented cationic PI-2DP with high selectivity and permeability in osmotic power generators, achieving a *P*_*max*_ of 40.4 W m^–2^ at 50-fold KCl, outperforming most state-of-the-art ion-selective membranes (Figure 8f).^32,61−64^ The power output (*P*_*out*_) can be exported by applying an external load resistance. For example, under 50-fold NaCl, PI-2DP demonstrates an excellent output power density, reaching a *P*_*out*_ of 48.4 W m^–2^ at 10^6^ Ω (Figure 8g). To evaluate their practical usage, we harnessed the energy between the Mediterranean seawater and the water from the Elbe River using PI-2DP, achieving an impressive *P*_*out*_ of 42.2 W m^–2^ (Figure 8h).

### 4.2 Electrode Coating in Metal Ion Batteries

**Figure 9:**
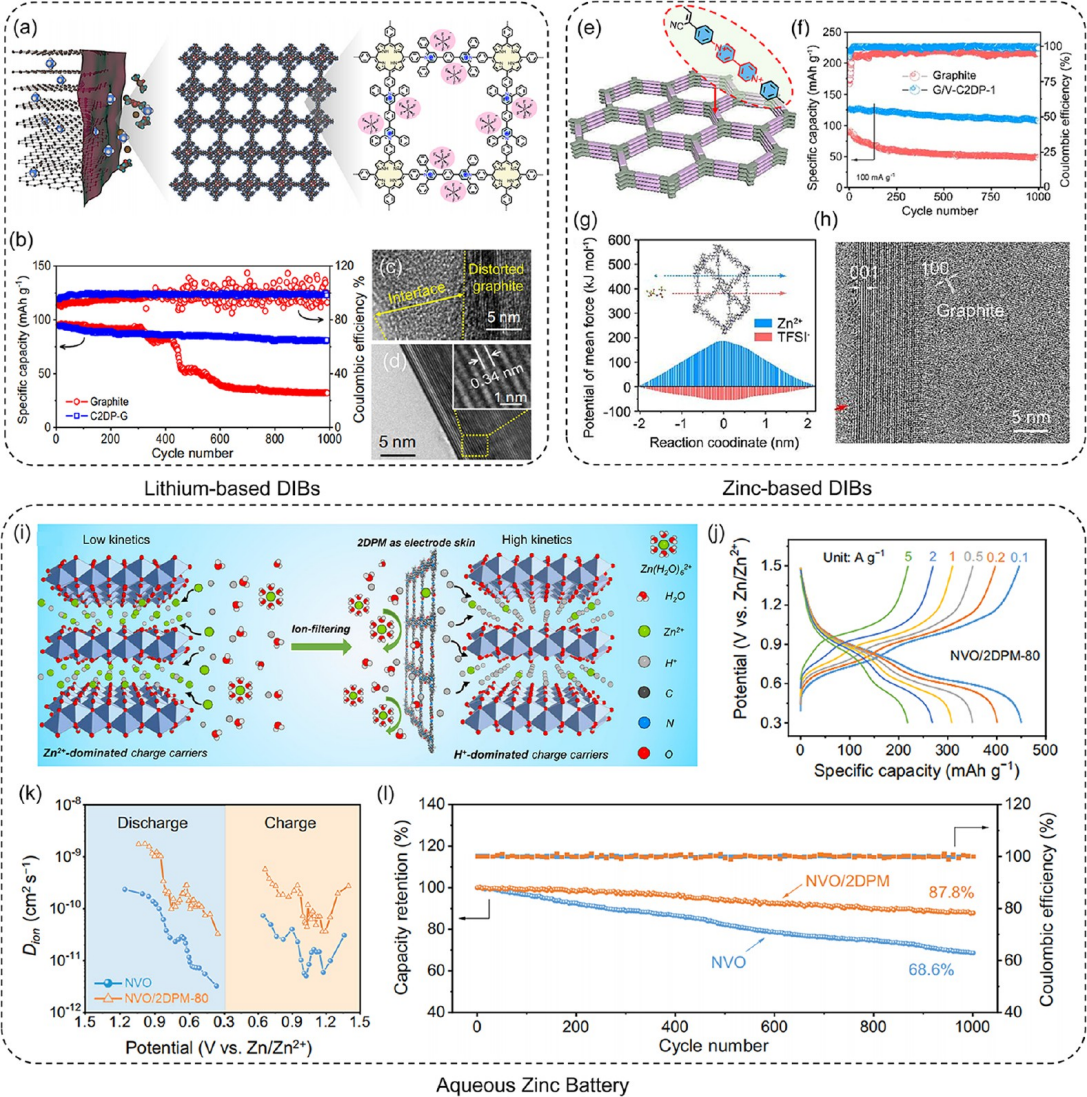
**Application of 2DPMs as electrode coating in metal ion batteries.** (a) Schematic of C2DP as an anion-interaction graphite electrode coating in lithium-based DIBs. (b) Cycling performance of the graphite and C2DP/G electrodes at 1C. TEM images of (c) graphite and (d) the C2DP/G electrodes. Reproduced from ref (4). Copyright 2023 Springer Nature. (e) Demonstration of V–C2DP as a graphite electrode coating in zinc-based DIBs. (f) Cycling performance of resulting zinc-based DIBs at 100 mA g^–1^. (g) Calculated Zn^2+^ and TFSI^–^ diffusion energy barriers through V–C2DP. (h) TEM image of V–C2DP/G electrode after 20 GCD cycles. Reproduced from ref (30). Copyright 2024 Wiley-VCH. (i) Schematic of the H^+^-dominated cathode coating enabled by 2DPI. (j) GCD curves of NVO/2DPI at various current densities. (k) Ion diffusion coefficient (*D*_*ion*_) of the NVO and NVO/2DPI at different potentials in AZBs. (l) Cycling performance of resulting AZBs based on NVO and NVO/2DPM at 3 A g^–1^. Reproduced from ref (33). Copyright 2024 Springer Nature.

Dual-ion batteries (DIBs) are emerging as a promising solution for high-voltage (>4.5 V), fast-charging, and large-scale energy storage applications, typically achieved by replacing the metal-ion-hosting cathodes (e.g., alkaline Li^+^, Na^+^, and multivalent Ca^2+^, Zn^2+,^ and Al^3+^) with anion-hosting graphite cathodes.^4,30^ However, DIBs encounter significant interfacial issues with anion-interaction graphite chemistries, including electrolyte decomposition, cation/solvent intercalations, and gas release, leading to low Colombia efficiency (CE) and potential device failure. To address these challenges, a common strategy involves applying a passivation layer on the anode surface with ideal ion-selective properties, allowing charge carrier ions to pass while insulating the electrolytes from the electrode. Recently, we introduced a positively charged 2D poly(pyridinium salt) membrane (C2DP) (pore size: 3 nm) as the coating for graphite electrodes in lithium-based DIBs (Figure 9a).^4^ Attributed to ultrathin thickness of 20 nm, high charge density, and well-defined 1D nanochannels, C2DP affords excellent anion-selective transport properties, with a PF_6_^–^ diffusivity of ∼10^–7^ cm^2^ s^–1^. Consequently, the C2DP-covered graphite (C2DP/G) cathode exhibits enhanced durability of PF_6_^–^ intercalation, retaining 92.8% of its capacity after 1000 cycles at 1 C, with CE exceeding 99%, which outperforms bare graphite electrode (CE decreased to 32% after 1000 cycle) (Figure 9b). HRTEM images of graphite electrodes show a distorted structure and thick interface due to electrolyte decomposition, whereas the C2DP/G electrode maintains a smooth surface and a highly crystalline structure after 20 galvanostatic charge/discharge (GCD) cycles (Figures 9c-d). These findings highlight the universal protective effect of C2DP for anion-intercalation chemistries.

In another study, we integrated a vinylene-linked cationic 2D polymer (V–C2DP) membrane with a face-on orientation, pore size of 2.2 nm, and thickness of 52 nm into a zinc-based DIB (Figure 9e).^30^ Utilizing its high anion selectivity and permeability (bis(trifluoromethanesulfonyl) imide (TFSI^–^) transport number up to 0.85), the V–C2DP-covered graphite (V–C2DP/G) cathode demonstrated improved specific capacity (124 mAh g^–1^ at 100 mA g^–1^), CE (92.90%), and cycling life with over 95% capacity retention (more than 1000 cycles), compared to bare graphite electrode (capacity: 54.2 mAh g^–1^ at 100 mA g^–1^, CE: 73.08%, capacity retention: 65% after 250 cycles) in zinc-based DIBs (Figure 9f). Relevant energy barriers for solvated Zn^2+^ and TFSI^–^ ions in the out-of-plane path of V–C2DP were calculated, revealing a notably higher diffusion barrier for Zn^2+^ (+187 kJ mol^–1^) compared to TFSI^–^ (−55 kJ mol^–1^), indicating the lower transport ability of Zn^2+^ in V–C2DP channels due to electrostatic repulsion (Figure 9g). This unique TFSI^–^-selectivity of V–C2DP prevents the formation of a dense electrolyte decomposition layer on the graphite electrode, effectively averting irreversible structural deformation (Figure 9h).

In addition to DIBs, the flourishing of 2DPMs presents opportunities for aqueous zinc batteries (AZBs), addressing challenges like limited storage capacity and durability due to sluggish Zn^2+^ kinetics as the primary charge carriers.^33^ The high H^+^/Zn^2+^ selectivity of 2DPI-80 allows it to function as an electrode coating, enabling rapid and selective proton conduction. This leads to a distinctive electrochemistry transition shifting from sluggish Zn^2+^-dominated to fast-kinetics H^+^-dominated Faradaic reactions (Figure 9i). By depositing 2DPI-80 onto the NaV_3_O_8_·1.5H_2_O electrode (denoted NVO/2DPI), an ultrahigh specific capacity of 450.5 mAh g^–1^ was achieved, representing a 56% capacity increase compared to bare NVO, approaching its theoretical limit (485.6 mAh g^–1^). Moreover, NVO/2DPI exhibited high-capacity retention of 50.7% with current density increasing from 0.1 to 5 A g^–1^ (Figure 9j). This high kinetics performance of NVO/2DPI is attributed to the enhanced charge storage dynamics resulting from accelerated proton diffusion (Figure 9k). Furthermore, NVO/2DPI batteries exhibit improved cycling stability, retaining 87.8% of their original capacity after 1000 GCD cycles with nearly 100% CE (Figure 9l).

## 5 Conclusions and Outlook

In this Account, we delineate our recent contributions in the synthesis of 2DPMs using the SMAIS method on the water surface, focusing on their selective-ion-transport properties and applications in sustainable energy devices. We first highlight the role of surfactants, the guided interactions between surfactants and monomer precursors, and the enhanced reactivity on the water surface. Additionally, the structure–property relationship of 2DPMs in selective ion transport has been elucidated, emphasizing factors including pore sizes, charged groups, crystallinity, and thickness. These milestones underscore the promising potential of 2DPMs in overcoming the trade-off between ion permeability and selectivity, contributing to superior selective-ion-transport properties. Finally, we survey the applications of 2DPMs in diverse energy devices, including osmotic power generators and metal ion batteries. Despite exciting achievements, several key fundamental issues require more attention in the years ahead, as outlined below.

(1) In synthetic chemistry, delving deeper into the mechanisms underlying 2D polymerization and crystallization on the water surface is essential, which will guide predictive synthesis efforts and facilitate the synthesis of 2DPMs with larger crystal domains. Refining SMAIS synthesis to yield high-quality ultrathin membranes or ideally monolayer single crystals, merits further investigation due to their potential to maximize ion selectivity while preserving high permeability. Also, expanding irreversible linkage chemistry (e.g., Suzuki coupling, Glaser coupling, and Aldol reactions) to produce robust 2DPMs with high chemical stability and mechanical strength is crucial for promoting their practical applications.
(2) From a materials science perspective, broadening the range of monomers with designed geometry, size, and ionic groups will endow 2DPMs with boosted selective-ion-transport properties. In this respect, the integration of machine learning via artificial intelligence holds great potential for expediting the discovery of innovative 2DPMs with optimized selective-ion-transport properties. Note that scaling up the production of 2DPMs remains challenging, which limits their practical usage. Reference to other polymeric membranes, the roll-to-roll technique represents a potentially feasible approach for the large-scale synthesis and transfer of high-quality 2DPMs.
(3) For ion transport studies and device applications, unraveling the impact of critical factors (e.g., pore size, charge density, thickness, defects, grain boundaries, and stacking modes) on their selective-ion-transport properties is paramount for establishing the structure–property relationship. To accomplish this, employing advanced characterization techniques (e.g., nuclear magnetic resonance (NMR) and advanced Terahertz (THz) techniques) across different spatiotemporal scales is essential to reveal the mechanisms of ion transport inside 2DPMs. For example, *ex-situ* techniques such as solid-state NMR (ssNMR)^65^ and pulsed-field gradient-stimulated-echo NMR (PFG-NMR)^66^ have been employed to determine the local ion movement by determining interactions between specific ions with functional groups of membranes. *In-situ* THz time-domain spectroscopy (THz-TDS) has also emerged as a powerful technique for probing the microscopic motion of ions in solid electrolytes.^67^ These techniques can offer detailed insights into the dynamics and interactions of ions within 2DPMs. Furthermore, achieving ultrahigh osmotic energy conversion in currently reported 2DPMs relies on a limited working area (typically at the micrometer level). Expanding this working area to an overmillimeter scale when maintaining the output power density remains challenging, necessitating the optimization of 2DPM quality and energy device configuration. Furthermore, exploring the integration of 2DPMs in fuel cells, electrolyzers, and various batteries, such as sodium-ions and potassium-ions batteries, is of significant interest. Finally, shifting the focus from purely laboratory-level assessments to real-application-oriented research is crucial for promoting their market competitiveness.
